# Supplementary figures and images for: The dependence of hydropower planning in relation to the influence of climate in Northeast Brazil
Source: PLoS One. 2022 Jan 25;17(1):e0259951. doi: 10.1371/journal.pone.0259951 (PMC8789118; doi:10.1371/journal.pone.0259951)

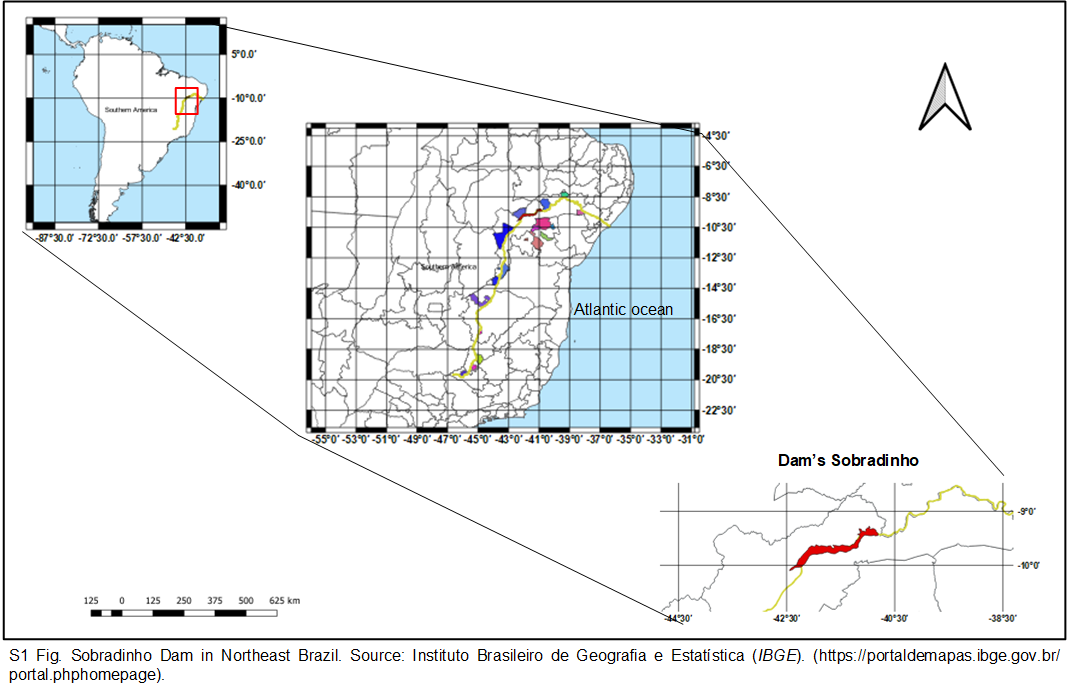

Supplement: S1 Fig — Source: Instituto Brasileiro de Geografia e Estatística (IBGE). (https://portaldemapas.ibge.gov.br/portal.phphomepage). (TIF) [file pone.0259951.s001.tif]

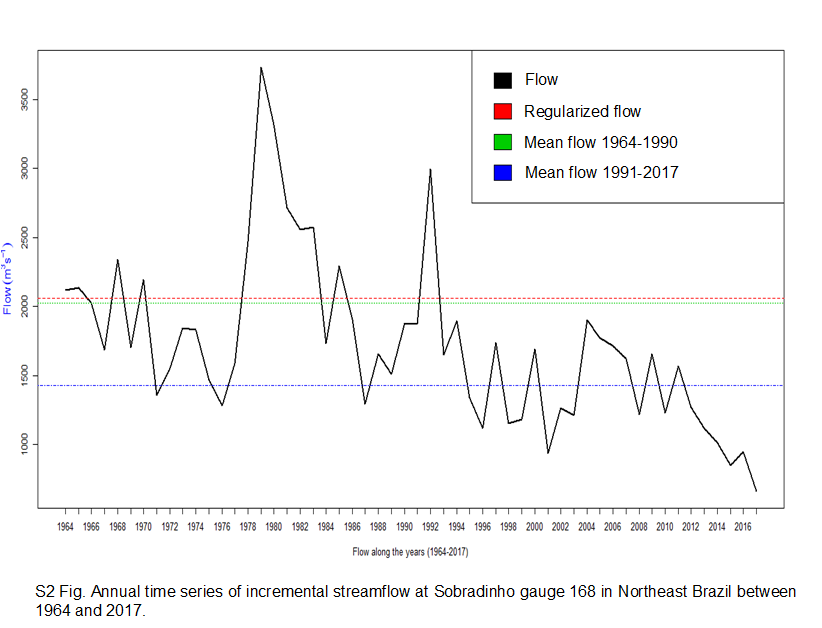

Supplement: S2 Fig — (TIF) [file pone.0259951.s002.tif]

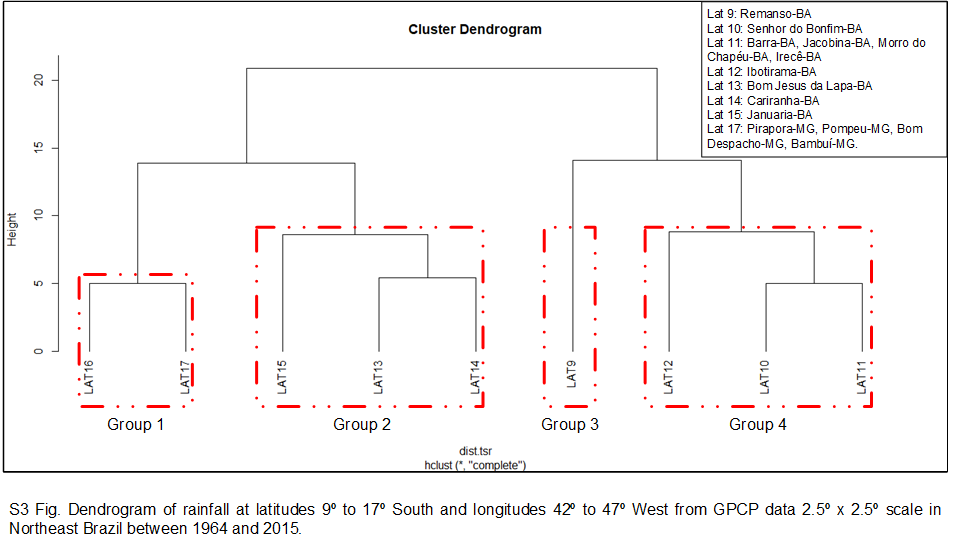

Supplement: S3 Fig — (TIF) [file pone.0259951.s003.tif]

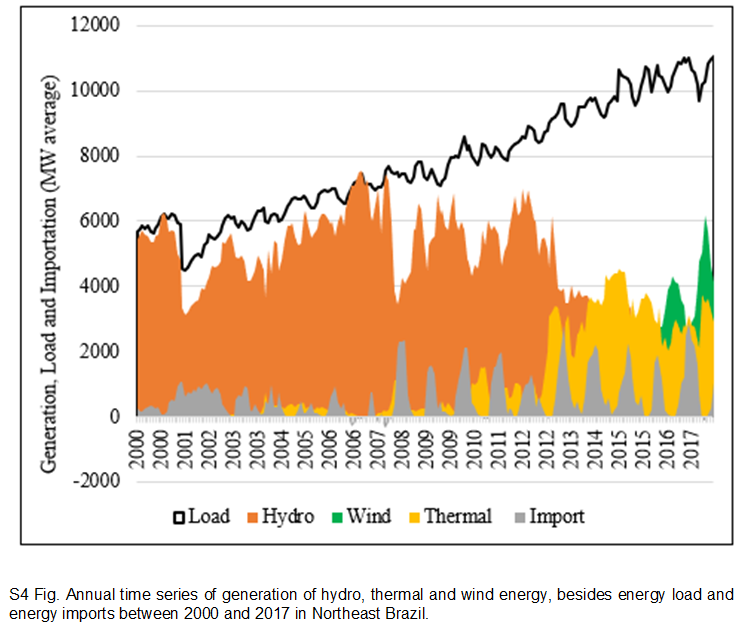

Supplement: S4 Fig — (TIF) [file pone.0259951.s004.tif]

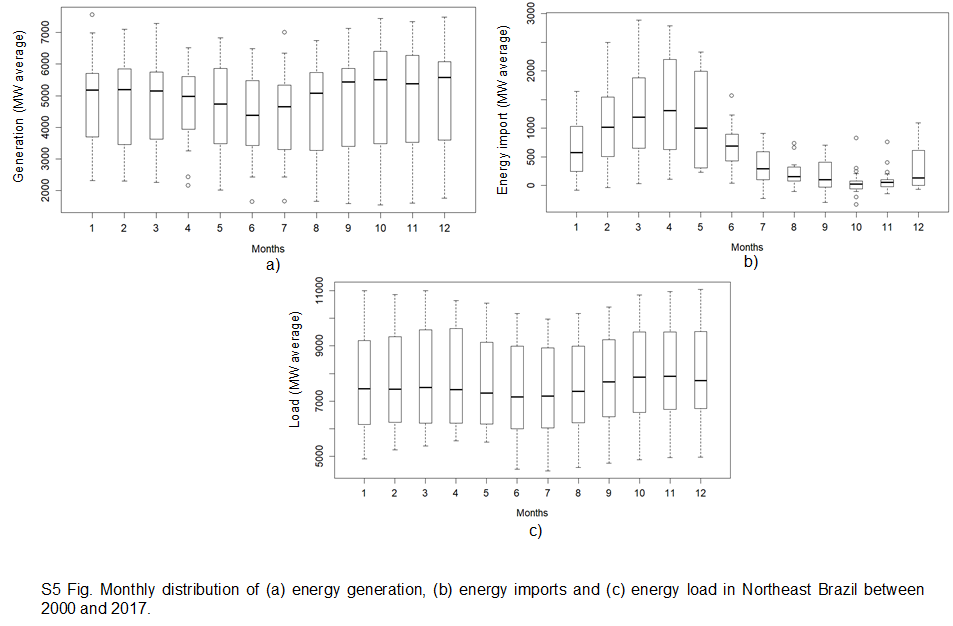

Supplement: S5 Fig — (TIF) [file pone.0259951.s005.tif]

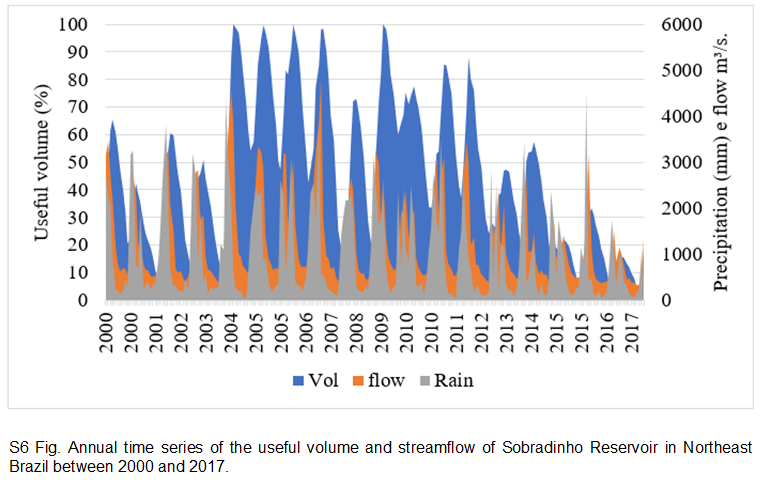

Supplement: S6 Fig — (TIF) [file pone.0259951.s006.tif]

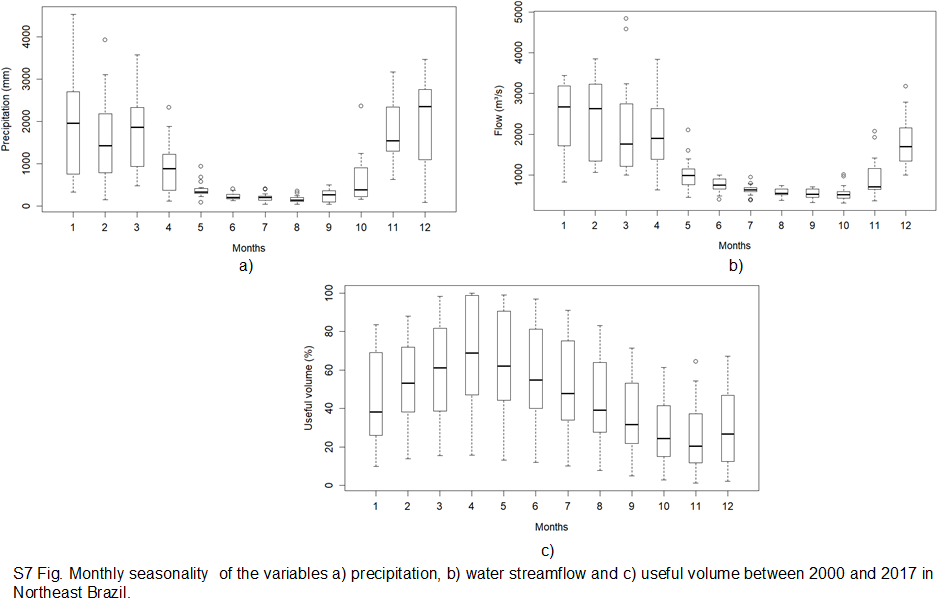

Supplement: S7 Fig — (TIF) [file pone.0259951.s007.tif]

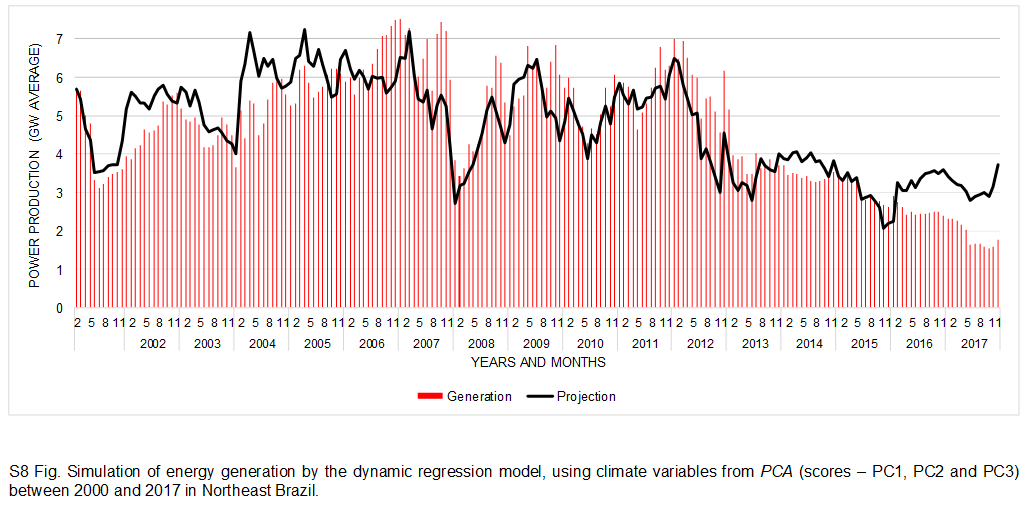

Supplement: S8 Fig — (TIF) [file pone.0259951.s008.tif]

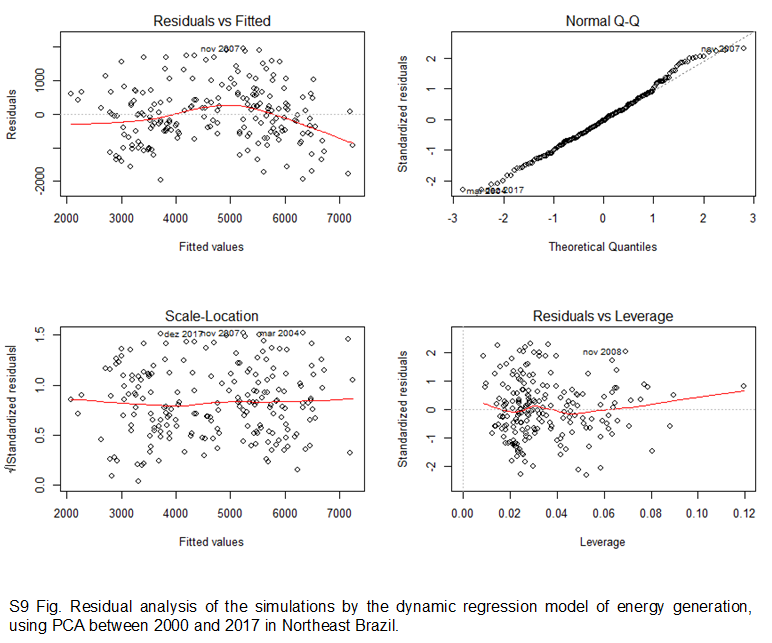

Supplement: S9 Fig — (TIF) [file pone.0259951.s009.tif]
