# Supplementary material for: The dependence of hydropower planning in relation to the influence of climate in Northeast Brazil
Source: PLoS One. 2022 Jan 25;17(1):e0259951. doi: 10.1371/journal.pone.0259951 (PMC8789118; doi:10.1371/journal.pone.0259951)
Supplement: S1 Table — (PDF) [file pone.0259951.s010.pdf]

S1 Table. Weather stations of *INMET*

City-State	Station	Latitude	Longitude	Altitude
Remanso-BA*	WMO 82979	9°6'S	42°1'W	400 m
Senhor do Bonfim-BA*	WMO 83088	10°4'S	40°1'W	558m
Barra-BA**	WMO 83179	11°S	43°1'W	401m
Jacobina-BA**	WMO 83186	11°18'S	40°4'W	484m
Morro do Chapéu-BA**	WMO 883184	11°2'S	41°21'W	1000m
Irecê-BA**	WMO 83182	11°3'S	41°86'W	747m
Bom Jesus da Lapa-BA**	WMO 83288	13°26'S	43°41'W	439m
Cariranha-BA**	WMO 83408	14°28'S	43°76'W	450m
Januária-BA**	WMO 83386	15°45'S	44°W	473m
Pirapora***	WMO 83483	17°35'S	44°9'W	505m
Pompeu-BA***	WMO 83570	19°21'S	45°W	691m
Bom Despacho-MG***	WMO 83533	19°68'S	45°36'W	695m
Bambuí-MG***	WMO 83582	20°03'S	45°W	661m

Lower São Francisco; **Middle São Francisco;*Upper São Francisco*

BA=Bahia;MG=Minas Gerais
