# Supplementary material for: The dependence of hydropower planning in relation to the influence of climate in Northeast Brazil
Source: PLoS One. 2022 Jan 25;17(1):e0259951. doi: 10.1371/journal.pone.0259951 (PMC8789118; doi:10.1371/journal.pone.0259951)
Supplement: S2 Table — All average values are in mm. (PDF) [file pone.0259951.s011.pdf]

Table 2. Descriptive statistics of rainfall in Northeast Brazil between 1964 and 2015 at latitudes 9° to 17° South and longitudes 42° to 47° West (2°.5' x 2°.5') with 5% significance level. All average values are in *mm*.

**Latitude**	**Periods**	**Average**	**b - a**	**statistic T**	**p-value**	**Mann-Kendall**	**p-value**
9°	1964-1989.a	50.34	-11.53	2.35	0.001	-0.899	0.013
	1990-2015.b	38.81					
10°	1964-1989.a	59.45	-5.42	0.97	0.327	-0.410	0.257
	1990-2015.b	54.03					
11°	1964-1989.a	68.48	-6.85	1.14	0.253	-0.594	0.100
	1990-2015.b	61.63					
12°	1964-1989.a	74.46	-9.96	1.63	0.100	-0.710	0.049
	1990-2015.b	64.50					
13°	1964-1989.a	73.80	-9.21	1.42	0.010	-0.466	0.196
	1990-2015.b	64.59					
14°	1964-1989.a	68.83	-8.00	1.21	0.224	-0.437	0.227
	1990-2015.b	60.83					
15°	1964-1989.a	74.13	-5.46	0.77	0.435	-0.326	0.366
	1990-2015.b	68.67					
16°	1964-1989.a	96.99	-7.66	0.92	0.357	-0.426	0.239
	1990-2015.b	89.33					
17°	1964-1989.a	103.12	-8.14	0.92	0.353	-0.489	0.175
	1990-2015.b	94.98					
