# Supplementary material for: The dependence of hydropower planning in relation to the influence of climate in Northeast Brazil
Source: PLoS One. 2022 Jan 25;17(1):e0259951. doi: 10.1371/journal.pone.0259951 (PMC8789118; doi:10.1371/journal.pone.0259951)
Supplement: S3 Table — Q*: Quarter. (PDF) [file pone.0259951.s012.pdf]

Table 3. Descriptive statistics of incremental streamflow ($m^3.s^{-1}$) at gauge 168 of Sobradinho Dam between 1964 and 2017 in Northeast Brazil. Q*: Quarter.

**Historic series**	**Average**			**Standard deviation**			**Variance**		
	64-90	91-17	64-17	64-90	91-17	64-17	64-90	91-17	64-17
1Q*	3,564	2,565	3,064	1,856	1,357	1,696	3,446	1,842	2,878
2Q*	1,840	1,299	1,570	1,062	790	971	1,128	624	944
3Q*	914	626	770	266	160	262	70	25	68
4Q*	1,790	1,224	1,507	985	772	926	970	596	859
