# Supplementary material for: The dependence of hydropower planning in relation to the influence of climate in Northeast Brazil
Source: PLoS One. 2022 Jan 25;17(1):e0259951. doi: 10.1371/journal.pone.0259951 (PMC8789118; doi:10.1371/journal.pone.0259951)
Supplement: S4 Table — (PDF) [file pone.0259951.s013.pdf]

Table 4. Correlation matrix R_{ij} of the original variables between 2000 and 2017 in Northeast Brazil and its weights.

**Variables**	**Ra**	**Flow**	**Useful volume**	**ASST-PAC**	**Dip**	$\hat{PC}_1$	$\hat{PC}_2$	$\hat{PC}_3$
**Ra**	1.000	0.59	0.005	-0.05	-0.04	0.88	-0.11	-0.11
**Flow**	0.590	1.000	0.440	-0.070	-0.070	0.85	0.35	0.00
**Useful Volume**	0.005	0.440	1.000	-0.090	0.010	0.23	0.79	0.26
**ASST-PAC**	-0.050	-0.070	-0.090	1.000	0.010	0.13	-0.64	0.47
**Dip**	-0.04	-0.07	0.010	0.040	1.000	0.11	0.00	0.84
