# Supplementary material for: The dependence of hydropower planning in relation to the influence of climate in Northeast Brazil
Source: PLoS One. 2022 Jan 25;17(1):e0259951. doi: 10.1371/journal.pone.0259951 (PMC8789118; doi:10.1371/journal.pone.0259951)
Supplement: S5 Table — (PDF) [file pone.0259951.s014.pdf]

Table 5. Summary of the dynamic regression model for energy generation, using the scores of the PCA between 2000 and 2017 in Northeast Brazil.

Model	Estimator	Value	Standard Error	t-value	p(> |t|)	R^2	Statistic F	p-value
1	Intercept	5,750.49	139.32	41.27	0.000	0.67	67.25	0.000
	G d6	0.281	0.072	3.90	0.000			
	G d13	0.368	0.059	6.17	0.000			
	Score PC1	134.61	63.53	2.11	0.010	***NSE***	***PBIAS***	***RMSE***
	Score PC2	594.51	62.04	9.58	0.000	0.67	0.00	846.68
	Score PC3	113.24	63.81	1.77	0.050			
	Trend G	-94.61	13.20	-7.163	0.000			
