# Supplementary material for: The dependence of hydropower planning in relation to the influence of climate in Northeast Brazil
Source: PLoS One. 2022 Jan 25;17(1):e0259951. doi: 10.1371/journal.pone.0259951 (PMC8789118; doi:10.1371/journal.pone.0259951)
Supplement: Attachment — Submitted filename: Response to reviewers_3.pdf [file pone.0259951.s023.pdf]

The dependence of the hydropower planning in relation to the influence of climate in the Northeast Brazil

Response to Reviewer 1

Reviewer correction

The point of reviewer: Topic 3: Title – "...title more specific to the study presented".

Answer

New title. "**The dependence of the hydropower planning in relation to the influence of Climate in the Northeast Brazil**". The change of the title intend to show a dependence between climate and hydropower generation.

Reviewer correction

The point of reviewer: Line 9-14: Sentence too long. Authors should be more concise.

Answer

The long sentence is due to the explanatory note of the acronym ERM – Energy Reallocation Mechanism. The idea is to consider and define how big problem of the dry period is in relation to the regulation of the electrical sector of ERM, when faced with drought. Also, because we can not to place footnote in the article (template). Anyway, we reduced.

Reviewer correction

The point of reviewer: Line 14-20: The text should rephrased.

Answer

This is the same case as the one previously mentioned. The text put the meaning of the both ERM and PG – Physical Guarantee, in parenthesis. Both terms could be in footnotes, but it is not allowed. Anyway, we reduced.

Reviewer correction

The point of reviewer: Line 20-26: The text should rephrased.

Answer

Considering the risks of ERM and the commitment to honor the physical guarantee, there is an inverse condition between water availability, the demand growth curve, in addition to the need to import energy and systemic restriction problems. The paragraph highlights the problem according to the context of the theme.

Reviewer correction

The point of reviewer: Line 297-299: The text should rephrased.

Answer

The text was rephrased.

Reviewer correction

The point of reviewer: Lines 37-38: A reference is necessary.

Answer

The references were placed.

Reviewer correction

The point of reviewer: Lines 43-47: Authors should briefly explain as these meteorological systems affect the area of study.

Answer

We made the summary about the meteorological systems between lines 49-53.

Reviewer correction

The point of reviewer: Lines 47-50: A reference is necessary.

Answer

The references were placed.

Reviewer correction – 3. Results and discussion

The point of reviewer:

1. To provide some information about will be expected in next decades.
2. Authors could use some local databases and relate bibliography to add this information.
3. To use NSE, PBIAS and RSR.

Answer

1. To provide some information about the future projection, we should make dynamic numeric model of the atmosphere-ocean to capture futures scenarios, but the aim of this paper was not make a projection, but a simulations with the past dataset, using dynamic regression model. Perhaps, it will be possible in a second step of the research.

2. This work did not aim to perform dynamic numerical simulation. Also, it did not have the character of exploring climate change.
3 . The NSE, PBIAS and RSR were incorporate into the study (Lines 202-205 and in table 4).

Reviewer correction – 4. Figures

The point of reviewer:

Point 4 – Figure 1: should be improved.

Point 4 – Figures: Figure 3 - About of the descriptive caption is the same of figure 2.

Answer

Figure 1 – The figure has been corrected and improved. The review figure is attached in the site.

Figure 3 - The figure caption has been corrected. The figure is attached in the site (Plos one).

Second step of review ↓

Reviewer correction – Line 10

The point of reviewer:

please, include a citation, and link if possible, to the ERM document.

Answer

To this term was make right citation [10] in the paper. ANEEL - https://www.aneel.gov.br/home?p_p_id=101&p_p_lifecycle=0&p_p_state=maximized&p_p_mode=view&_101_struts_action=%2Fasset_publisher%2Fview_content&_101_returnToFullPageURL=%2F&_101_assetEntryId=15050507&_101_type=content&_101_groupId=656835&_101_urlTitle=mecanismo-de-realocacao-de-energia-mre

Reviewer correction – Line 20-22

The point of reviewer:

A parenthesis is missing.

Answer

The parenthesis were placed.

Reviewer correction – Line 35-39

The point of reviewer:

The ITCZ This part of the text is confusing. Please, explain it better.

Answer

We tried to improve the text by adding a small note highlighting the hydro model versus water scarcity.

Reviewer correction – Line 42-43

The point of reviewer:

The ITCZ and the SACZ are climatological features, not meteorological systems. Please, fix it.

Answer

To avoid doubth about this concept, the term was change to atmospheric system.

Reviewer correction – Line 47

The point of reviewer:

March, April.

Answer

Both the first letters of March and April were replaced by capital letters.

Reviewer correction – Line 50-53

The point of reviewer: You should make more explicit the link here between the exposed phenomena and water availability in the region of study (or rainfall). Values of correlations, percentage of water attributable to each phenomenon, etc. In general, I miss a complete introductory discussion here on this issue.

Answer

We tried to improve the text by adding a small note in addition, only to load attention to the future projections in the studied region with rainfall based.

Reviewer correction – Line 69

The point of reviewer:

water storage? I wonder how you translate the potential data to 'energy' stored

Answer

This is a simple concept and much used at electrical sector. As from storage energy it is possible to calculated its, due the producible energy by useful volume storage in the dam on its operatives levels. The National Operator of electric system from Brazil available all database about this parameters, separated by region. Source: http://www.ons.org.br/Paginas/resultados-da-operacao/historico-da-operacao/energia_armazenada.aspx . The INMET has already published, for example, low level warning of dams in 2021 - https://portal.inmet.gov.br/noticias/sistema-nacional-de-meteorologia-snm-emite-alerta-de-emergência-hídrica.

Reviewer correction – l234, l237, l240, l259, l294 (and any other along the manuscript)

The point of reviewer:

Avoid using the 'surname et al.' citation style. You could use 'According to existing research [34]'

Answer

All this situations in the manuscript that were used “author et al.”, have been fix as recommended.

Reviewer correction – Data Inmet and streamflow

The point of reviewer:

The data section does not contain enough information on your data sources. During the submission, you have included in the 'Data availability section links to climatological indexes. However, there is no information about getting the rainfall data from the INMET or where a reader can access it. The same happens with the incremental streamflow data (line 121). Please, all the data must be accessible and available to anyone. Include the relevant links and, if possible, the DOI of the dataset. You could upload the data to Zenodo to make them available and get a DOI.

Answer

The data of INMET (www.inmet.gov.br) and incremental streamflow are available and attached in the site (Plos one).

Reviewer correction – Conclusion

The point of reviewer:

In the conclusions: It is confusing how to move from a 5000 MW deficit to 2000 MW is a worse scenario. It is necessary a better explanation here. Also, the statement on the need to increase energy imports is out of scope here. Please, remove it. This manuscript is not about the socioeconomics of the issue, and recommendations on the best strategy to deal with deficits of a power system would be a different discussion, involving studies on other possibilities, technologies, etc.

Answer

It is possible observe that in the last years the supply of hydropower has been decreasing and to solve in the short time it is necessary to energy import from others regions of Brazil. I am not understanding what relation between socioeconomics and energy import.
