# Supplementary material for: The dependence of hydropower planning in relation to the influence of climate in Northeast Brazil
Source: PLoS One. 2022 Jan 25;17(1):e0259951. doi: 10.1371/journal.pone.0259951 (PMC8789118; doi:10.1371/journal.pone.0259951)
Supplement: Attachment — Submitted filename: Reviewer 1_PLOS ONE_Detailed Answers.docx [file pone.0259951.s022.docx]

**Climate and hidropower planning in the Northeast Brazil**

**Response to Reviewer 1**

**Reviewer correction**

 The point of reviewer: Topic 3: Title – “…title more specific to the study presented”.

**Answer**

New title. “**The dependence of the hydropower planning in relation to the influence of Climate in the Northeast Brazil**“. The change of the title intend to show a dependence between climate and hydropower generation.

Short title: Hydropower crisis and the climate.

**Reviewer correction**

The point of reviewer: Line 9-14: Sentence too long. Authors should be more concise.

**Answer**

The long sentence is due to the explanatory note of the acronym ERM – Energy Reallocation Mechanism. The idea is to consider and define how big problem of the dry period is in relation to the regulation of the electrical sector of ERM, when faced with drought. Also, because we can not to place footnote in the article (template). Anyway, we reduced.

**Reviewer correction**

 The point of reviewer: Line 14-20: The text should rephrased.

**Answer**

This is the same case as the one previously mentioned. The text put the meaning of the both ERM and PG – Physical Guarantee, in parenthesis. Both terms could be in footnotes, but it is not allowed. Anyway, we reduced.

**Reviewer correction**

 The point of reviewer: Line 20-26: The text should rephrased.

**Answer**

Considering the risks of ERM and the commitment to honor the physical guarantee, there is an inverse condition between water availability, the demand growth curve, in addition to the need to import energy and systemic restriction problems. The paragraph highlights the problem according to the context of the theme.

**Reviewer correction**

 The point of reviewer: Line 297-299: The text should rephrased.

**Answer**

The text was rephrased.

**Reviewer correction**

The point of reviewer: Lines 37-38: A reference is necessary.

**Answer**

The references were placed.

**Reviewer correction**

The point of reviewer: Lines 43-47: Authors should briefly explain as these meteorological systems affect the area of study.

**Answer**

We made the summary about the meteorological systems between lines 49-53.

**Reviewer correction**

The point of reviewer: Lines 47-50: A reference is necessary.

**Answer**

The references were placed.

**Reviewer correction – 3. Results and discussion**

The point of reviewer:

1. To provide some information about will be expected in next decades.
2. Authors could use some local databases and relate bibliography to add this information.
3. To use NSE, PBIAS and RSR.

**Answer**

1. To provide some information about the future projection, we should make dynamic numeric model of the atmosphere-ocean to capture futures scenarios, but the aim of this paper was not make a projection, but a simulations with the past dataset, using dynamic regression model. Perhaps, it will be possible in a second step of the research.

2. This work did not aim to perform dynamic numerical simulation. Also, it did not have the character of exploring climate change.

3 . The NSE, PBIAS and RSR were incorporate into the study (Lines 202-205 and in table 4).

**Reviewer correction – 4. Figures**

The point of reviewer:

Point 4 – Figure 1: should be improved.

Point 4 – Figures: Figure 3 - About of the descriptive caption is the same of figure 2.

**Answer**

Figure 1 – The figure has been corrected and improved. The review figure is attached in the site.

Figure 3 - The figure caption has been corrected. The figure is attached in the site (Plos one).
